# Cost-effectiveness of a *Wolbachia*-based replacement strategy for dengue control in Brazil

**DOI:** 10.1016/j.lana.2024.100789

**Published:** 2024-05-24

**Authors:** Hugo C. Turner

**Affiliations:** MRC Centre for Global Infectious Disease Analysis, School of Public Health, Imperial College London, London, UK

Dengue is a mosquito-borne arboviral pathogen that is widespread in tropical and sub-tropical climates worldwide. The global incidence of dengue has grown dramatically with approximately half of the world's population now at risk.^1^

Globally, dengue is responsible for a notable health and economic burden and preventive interventions to control it are vital. One such intervention is the release of *Aedes aegypti* mosquitoes infected with the intracellular bacterium *Wolbachia*. These *Wolbachia-*infected mosquitoes can either be used to replace the existing mosquito population with one less likely to transmit infection generating long-term reductions in transmission (a *Wolbachia*-based population replacement approach) or to suppress the existing mosquito population by releasing males only (a *Wolbachia*-based population suppression approach).

The implementation of this intervention has been gradually increasing in scale. For example, the World Mosquito Program has implemented releases of *Wolbachia-*infected mosquitoes across the cities of Bello, Medellín and Itagüí in Colombia, covering an area of 135 km^2^, and a combined population of 3.3 million people.^2^

In *The Lancet Regional Health—Americas*, Zimmermann et al.^3^ conducted an economic evaluation to simulate the potential cost-effectiveness of the implementation of a *Wolbachia*-based population replacement strategy covering seven cities in Brazil. For this, they developed an individual-based microsimulation State Transition Model to simulate nearly 23 million individuals over a 20-year period. The analysis adopted a societal perspective, considering both direct and indirect costs (also known as productivity costs) associated with dengue cases. The reference scenario of the routine dengue control activities carried out by the public health authorities was used as the comparator.

The model projected that adding the implementation of a *Wolbachia*-based population replacement strategy to the current routine dengue control strategies would lead to a significant reduction in the incidence of dengue cases. When taking a societal perspective, the intervention was projected to generate cost-savings compared to the reference scenario in each of the seven targeted cities; i.e. the long-term reduction in the costs associated with dengue cases outweighs the cost of the initial intervention. Notably, the averted productivity costs were a key driver in these savings.

The positive findings were projected to be robust within the sensitivity analysis. For example, when shorter time horizons were considered (5 or 10 years of benefits), the intervention was still projected to generate cost savings although the magnitude of the savings was lower when compared to the baseline 20-year time horizon. It should be noted that when the analysis used a narrower perspective (no longer considering averted productivity costs), the intervention was no longer projected to generate cost savings in every city investigated, although the projected incremental cost-effectiveness ratios remained under the considered cost-effectiveness threshold. Similar findings were reported by a study investigating the cost-effectiveness of the *Wolbachia*-based population replacement strategy in the Brazilian state of Goiás.^3^

Overall, the study adds to the growing evidence base that *Wolbachia*-based population replacement interventions can be a cost-effective way of controlling dengue. This supports the results of studies relating to other settings (including Indonesia,^4^ Thailand,^5^ and Vietnam^6^). This evidence base is relevant for policymakers and supports the use of *Wolbachia-*based interventions when they are targeted to high-burden settings—particularly high-density urban environments.

That said, it is important to consider that important research gaps remain in this area which require further investigation. For example, the impact of *Wolbachia-*based interventions can be heterogeneous and influenced by local environmental factors.^7^^,^^8^ Further work is needed to better understand this potential variation and to better account for it when making long-term projections of the impact of *Wolbachia-*based interventions—particularly in the context of climate change.^9^ There is also a need to further evaluate the use of *Wolbachia* interventions in combination with other preventive dengue interventions. This is now particularly pertinent with new vaccines becoming available.^10^ In this context, it is important to consider that although *Wolbachia-*based interventions can be a cost-effective (and even cost-saving) intervention, it does not mean it is cost-effective universally across endemic areas—particularly in more rural areas. There is, therefore, an ongoing need to investigate the combination of *Wolbachia-*based interventions and dengue vaccination more comprehensively—considering the use of different interventions in different areas within countries.

## Contributors

HCT solely conceptualised this article, wrote the original draft, and reviewed and edited the final submitted draft.

## Declaration of interests

HCT has previously received funding from the World Mosquito Program.
